# Predictors of cardiac involvement in idiopathic inflammatory myopathies

**DOI:** 10.3389/fimmu.2023.1146817

**Published:** 2023-03-08

**Authors:** Matilde Bandeira, Eduardo Dourado, Ana Teresa Melo, Patrícia Martins, Vanessa Fraga, José Luís Ferraro, André Saraiva, Marlene Sousa, Hugo Parente, Catarina Soares, Ana Margarida Correia, Diogo Esperança Almeida, Sara Paiva Dinis, Ana Sofia Pinto, Filipe Oliveira Pinheiro, Maria Seabra Rato, Tiago Beirão, Beatriz Samões, Bernardo Santos, Carolina Mazeda, Ana Teodósio Chícharo, Margarida Faria, Agna Neto, Maria Helena Lourenço, Luísa Brites, Marília Rodrigues, Joana Silva-Dinis, João Madruga Dias, Filipe C. Araújo, Nádia Martins, Maura Couto, Ana Valido, Maria José Santos, Sofia Carvalho Barreira, João Eurico Fonseca, Raquel Campanilho-Marques

**Affiliations:** ^1^ Serviço de Reumatologia, Centro Hospitalar Universitário Lisboa Norte, Centro Académico de Medicina de Lisboa (CAML), Lisboa, Portugal; ^2^ Unidade de Investigação em Reumatologia, Instituto de Medicina Molecular, Faculdade de Medicina, Universidade de Lisboa, CAML, Lisboa, Portugal; ^3^ Serviço de Reumatologia, Hospital Garcia de Orta, Almada, Portugal; ^4^ Serviço de Reumatologia, Hospital Beatriz Ângelo, Loures, Portugal; ^5^ Serviço de Reumatologia, Centro Hospitalar Universitário de Coimbra, Coimbra, Portugal; ^6^ Serviço de Reumatologia, Unidade Local de Saúde do Alto Minho, Ponte de Lima, Portugal; ^7^ Serviço de Reumatologia, Hospital de Braga, Braga, Portugal; ^8^ Serviço de Reumatologia, Unidade Local de Saúde da Guarda, Guarda, Portugal; ^9^ Serviço de Reumatologia, Centro Hospitalar Universitário de São João, Porto, Portugal; ^10^ Serviço de Reumatologia, Centro Hospitalar de Vila Nova de Gaia/Espinho, Vila Nova de Gaia, Portugal; ^11^ Serviço de Reumatologia, Centro Hospitalar do Baixo Vouga, Aveiro, Portugal; ^12^ Serviço de Reumatologia, Centro Hospitalar Universitário do Algarve, Faro, Portugal; ^13^ Serviço de Reumatologia, Hospital Nélio Mendonça, Serviços de Saúde da Região Autónoma da Madeira, Funchal, Portugal; ^14^ Serviço de Reumatologia, Centro Hospitalar de Lisboa Ocidental, Lisboa, Portugal; ^15^ Serviço de Reumatologia, Centro Hospitalar de Leiria, Leiria, Portugal; ^16^ Serviço de Reumatologia, Centro Hospitalar Universitário de Lisboa Central, Lisboa, Portugal; ^17^ Serviço de Reumatologia, Centro Hospitalar de Médio Tejo, Tomar, Portugal; ^18^ Serviço de Reumatologia, Hospital CUF Cascais, Cascais, Portugal; ^19^ Serviço de Reumatologia, Centro Hospitalar Tondela-Viseu, Viseu, Portugal; ^20^ Serviço de Reumatologia, Unidade Local de Saúde do Litoral Alentejano, Santiago do Cacém, Portugal

**Keywords:** idiopathic inflammatory myopathies, cardiac involvement, myocarditis, risk factors, biomarkers, predictors

## Abstract

**Objectives:**

Idiopathic inflammatory myopathies (IIM) are a group of rare disorders that can affect the heart. This work aimed to find predictors of cardiac involvement in IIM.

**Methods:**

Multicenter, open cohort study, including patients registered in the IIM module of the Rheumatic Diseases Portuguese Register (Reuma.pt/Myositis) until January 2022. Patients without cardiac involvement information were excluded. Myo(peri)carditis, dilated cardiomyopathy, conduction abnormalities, and/or premature coronary artery disease were considered.

**Results:**

230 patients were included, 163 (70.9%) of whom were females. Thirteen patients (5.7%) had cardiac involvement. Compared with IIM patients without cardiac involvement, these patients had a lower bilateral manual muscle testing score (MMT) at the peak of muscle weakness [108.0 ± 55.0 vs 147.5 ± 22.0, p=0.008] and more frequently had oesophageal [6/12 (50.0%) vs 33/207 (15.9%), p=0.009] and lung [10/13 (76.9%) vs 68/216 (31.5%), p=0.001] involvements. Anti-SRP antibodies were more commonly identified in patients with cardiac involvement [3/11 (27.3%) vs 9/174 (5.2%), p=0.026]. In the multivariate analysis, positivity for anti-SRP antibodies (OR 104.3, 95% CI: 2.5-4277.8, p=0.014) was a predictor of cardiac involvement, regardless of sex, ethnicity, age at diagnosis, and lung involvement. Sensitivity analysis confirmed these results.

**Conclusion:**

Anti-SRP antibodies were predictors of cardiac involvement in our cohort of IIM patients, irrespective of demographical characteristics and lung involvement. We suggest considering frequent screening for heart involvement in anti-SRP-positive IIM patients.

## Introduction

Idiopathic inflammatory myopathies (IIM) are rare systemic rheumatic disorders that primarily affect the muscle, joints, skin, and lungs in varying degrees. Although less often recognised, cardiovascular complications represent a major cause of death (1).

Myocarditis is a classic and severe but uncommon manifestation of IIM (2), while conduction abnormalities and diastolic dysfunction are more common (3, 4). Usually asymptomatic and not life-threatening, pericarditis is reported in 4-25% of IIM patients (5). Pulmonary hypertension appears to be mostly secondary to interstitial lung disease (ILD) and unlikely in the absence of antisynthetase antibodies (5). An increased incidence of accelerated atherosclerosis is also recognised in most rheumatic inflammatory diseases, including IIM (5).

Inflammation similar to that found in skeletal muscle is seen in both the heart muscle (3, 6) and the conduction system (6) in autopsies of IIM patients. Additionally, magnetic resonance imaging (MRI) studies showed an association between myocardial enhancement on MRI and diastolic dysfunction (7). These studies suggest that myocardial inflammation is a primary driver of heart dysfunction and conduction abnormalities in IIM.

Despite the recognition of several forms of cardiac involvement in IIM, specific guidelines for the screening, diagnosis, treatment, and follow-up are still lacking (8). An important first step towards the definition of optimal screening strategies is the identification of clinically useful predictors.

A large multicenter study reported that smoking was more prevalent in patients with cardiac involvement (9). However, smoking was also more frequent in patients with ILD, and no multivariate analysis was performed to evaluate whether it was an independent predictor of cardiac involvement, adjusted for the presence of ILD (9). It has also been reported that patients with anti-Mi2 antibodies more frequently have chest pain than IIM patients without any myositis-specific antibody (MSA), despite having no known cardiovascular disease (10). Finally, there have been reports, although sometimes contradictory (10, 11), of an association between cardiac involvement and the presence of anti-signal recognition particle (SRP) antibodies (12, 13). However, the only study with a large cohort that confirmed this association did not exclude the possible influence of other confounders (13).

Given the currently identified clinical unmet need, this work aimed to identify predictors of cardiac involvement in IIM patients.

## Methods

This study is a multicenter cohort study, including patients registered in the IIM module of the Rheumatic Diseases Portuguese Register (14) (Reuma.pt/Myositis) from April 2019 until January 2022. The study is based on a retrospective analysis of prospectively collected data. Patients without information regarding the presence or absence of cardiac involvement were excluded. After data extraction, key missing data were identified and retrieved from the patients’ local clinical files.

Cardiac involvement was defined as myocarditis, pericarditis, dilated cardiomyopathy, conduction abnormalities, and/or premature coronary artery disease occurring due to IIM, according to the physician’s clinical judgement. Of note, cancer-associated myositis was defined as the onset of IIM within three years of a cancer diagnosis (15).

All patients were followed within the context of a Rheumatology department in a tertiary centre, and all cases of cardiac involvement were reviewed by the authors. The patients were screened for anti-nuclear antibodies (ANA) with indirect immunofluorescence assay (IIFA) on HEp-2 cells, MSA and myositis-associated autoantibodies (MAA) status by EUROLINE Autoimmune Inflammatory Myopathies 16 Ag (IgG) immunoblot.

Descriptive statistics were presented as median ± interquartile range for continuous variables and absolute and relative frequencies for categorical variables. Univariate analysis was performed using chi-square, Fisher’s exact, Mann-Whitney or t-test, as appropriate. Patients without cardiac involvement were used as a control group for this analysis. Predictors of cardiac involvement, adjusted for sex and age at diagnosis, were identified through binomial logistic regression modelling. The linearity of the continuous variables with respect to the logit of the dependent variable was assessed *via* the Box-Tidwell test. Correlated variables and cases with missing information were excluded from the multivariate analysis in order to fulfil all assumptions necessary to ensure the validity of the regression. Sensitivity analysis was performed by developing additional binomial logistic models. The statistical analysis was performed with SPSS/IBM version 23. The Bonferroni correction for multiple comparisons was applied to get α<0.05. Accordingly, in the univariate analysis, definite associations were defined by p<0.004 (marked with **) and likely associations by p<0.05 (marked with *).

The study was conducted according to the principles of the Declaration of Helsinki and was approved by the Ethics Committee of *Centro Académico de Medicina de Lisboa* (195/21). Included patients signed the Reuma.pt informed consent and data were fully anonymised throughout the entire research process. Reuma.pt is approved by the national data protection board (*Comissão Nacional de Proteção de Dados*) and by the Ethics Committee of all participating centres.

## Results

In this study, 230 patients were included. Over two-thirds of the patients (N=163/230, 70.9%) were females. Most patients were Caucasians (N=105/117, 89.7%), but African (N=11/117, 9.4%) and Asian (N=1/117, 0.9%) ancestries were also represented. The median age at disease onset was 49.0 ± 25.0 years, and the median age at diagnosis was 50.1 ± 25.0 years. The median disease duration at the last follow-up was 4.8 ± 6.5 years.

Thirteen patients (5.7%) had cardiac involvement, some with more than one feature. Six patients (2.6%) had clinically evident myocarditis, five (2.2%) had conduction abnormalities, three (1.3%) had dilated cardiomyopathy, two (0.9%) had pericarditis, and two (0.9%) had premature coronary artery disease.

In our cohort, Caucasians were less likely to have cardiac involvement than other ethnicities (p<0.001**). Among patients with cardiac involvement, four (57.1%) were Caucasian, two (28.6%) had African ancestry, and one (14.3%) had Asian ancestry. On the other hand, patients without heart involvement were predominantly Caucasian (91.8%), and 8.2% had African ancestry. Regarding sex, age at diagnosis, and other demographic variables, as well as smoking and alcohol intake habits, there were no statistically significant differences between the groups (**Table 1**). Mortality was also not significantly different between both groups [1/13 (7.7%) vs 9/217 (4.1%), p=0.448]. Of note, patients with cardiac involvement had a significantly shorter disease duration than controls [1.4 ± 0.9 vs 6.1 ± 5.7 years, p=0.003**].

**Table 1:** Clinical and serological features of patients with and without cardiac involvement.

	Patients with heart involvement (n=13)	Patients without heart involvement (n=217)	Univariate analysis
Age at diagnosis, median ± IQR (N)	52.2 ± 14.0 (13)	47.0 ± 24.0 (188)	p=0.079
**Disease duration (in years), median ± IQR (N)**	**1.4 ± 0.9 (13)**	**6.1 ± 5.7 (192)**	p=0.003**
Female, n/N (%)	8/13 (61.5)	155/217 (71.4)	p=0.531
Mortality, n/N (%)	1/13 (7.7)	9/217 (4.1)	p=0.448
Clinical data			
Musculoskeletal involvement			
Proximal muscle weakness, n/N (%)	9/13 (69.2)	144/175 (82.3)	p=0.267
Myositis, n/N (%)	11/13 (84.6)	174/212 (82.1)	p=1.000
**Minimum MMT8, median ± IQR (N)**	**108.0 ± 55.0 (7)**	**147.5 ± 22.0 (125)**	p=0.008*
Arthritis, n/N (%)	4/7 (57.1)	30/86 (34.9)	p=0.254
Skin involvement			
Gottron’ sign, n/N (%)	2/12 (16.7)	68/152 (44.7)	p=0.072
Heliotrope rash, n/N (%)	5/13 (38.5)	92/215 (42.8)	p=1.000
Gottron’s papules, n/N (%)	2/13 (15.4)	84/213 (39.4)	p=0.138
Malar rash, n/N (%)	2/9 (22.2)	28/121 (23.1)	p=1.000
**Subcutaneous oedema, n/N (%)**	**6/13 (46.2)**	**41/210 (19.5)**	p=0.034*
Shawl sign, n/N (%)	1/9 (11.1)	25/120 (20.8)	p=0.686
Mechanic’s hands, n/N (%)	4/9 (44.4)	20/120 (16.7)	p=0.061
Calcinosis, n/N (%)	0/12 (0.0)	21/215 (9.8)	p=0.609
Highest modified DAS skin, median ± IQR (N)	0.0 ± 3.0 (10)	1.0 ± 4.0 (122)	p=0.490
Internal organ involvement			
**Lung involvement, n/N (%)**	**10/13 (76.9)**	**68/216 (31.5)**	p=0.001**
**Esophageal involvement, n/N (%)**	**6/12 (50.0)**	**33/207 (15.9)**	p=0.009*
Systemic involvement			
**Weight loss, n/N (%)**	**4/8 (50.0)**	**17/118 (14.4)**	p=0.026*
Fever, n/N (%)	1/9 (11.1)	5/118 (4.2)	p=0.363
Neoplasia, n/N (%)	0/8 (0.0)	12/118 (10.2)	p=1.000
Complementary diagnostic exams			
Myopathic pattern on electromyogram, n/N (%)	8/10 (80.0)	88/122 (72.1)	p=0.727
Muscle oedema (STIR) on MRI, n/N (%)	1/2 (50.0)	30/58 (51.7)	p=1.000
Antibodies			
ANA, n/N (%)	10/13 (76.9)	141/205 (68.8)	p=0.759
SSA/SSB, n/N (%)	4/12 (33.3)	59/197 (29.9)	p=0.756
Anti-RNP, n/N (%)	0/12 (0.0)	11/196 (5.6)	p=1.000
Anti-Mi2, n/N (%)	1/12 (8.3)	26/184 (14.1)	p=1.000
Anti-TIF1γ, n/N (%)	1/11 (9.1)	5/172 (2.9)	p=0.314
Anti-MDA5, n/N (%)	1/11 (9.1)	8/172 (4.7)	p=0.435
Anti-NXP2, n/N (%)	1/11 (9.1)	5/170 (2.9)	p=0.317
Anti-SAE1, n/N (%)	0/11 (0.0)	6/170 (3.5)	p=1.000
**Anti-SRP, n/N (%)**	**3/11 (27.3)**	**9/174 (5.2)**	p=0.026*
Anti-Jo1, n/N (%)	4/12 (33.3)	47/201 (23.4)	p=0.487
Anti-PL7, n/N (%)	1/11 (9.1)	7/180 (3.9)	p=0.384
Anti-PL12, n/N (%)	1/11 (9.1)	6/180 (3.3)	p=0.344
Anti-EJ, n/N (%)	1/11 (9.1)	3/174 (1.7)	p=0.219
Anti-OJ, n/N (%)	1/11 (9.1)	2/173 (1.2)	p=0.170
Antysynthetase antibodies, n/N (%)	6/12 (50.0)	58/202 (28.7)	p=0.110
Anti-Pm/Scl, n/N (%)	0/12 (0.0)	16/186 (8.6)	p=0.604
Anti-Ku, n/N (%)	0/12 (0.0)	9/178 (5.1)	p=1.000

According to Bonferroni’s correction for multiple testing, variables were considered as likely associations (*) or definite associations (**). n, number of patients positive for the variable of interest; N, number of patients without missing information regarding the variable of interest; MMT8, manual muscle testing; DAS, disease activity score; STIR, short tau inversion recovery; MRI, magnetic resonance imaging; ANA, anti-nuclear antibodies; SSA/SSB, anti,Sjögren’s syndrome-related antigen A/B; RNP, ribonucleoprotein; TIF1γ, transcription intermediary factor 1-gamma; MDA5, melanoma differentiation-associated gene 5; NXP2, nuclear matrix protein 2; SAE, small ubiquitin-like modifier activating enzyme; SRP, signal recognition particle; Jo1, histidyl tRNA synthetase; PL7, threonyl tRNA synthetase; PL12, anti-alanyl tRNA synthetase; EJ, glycyl tRNA synthetase; OJ, isoleucyl tRNA synthetase; Pm/Scl, polymyositis/scleroderma.All statistically significant differences between groups are shown in bold.

Patients with cardiac involvement had a lower bilateral manual muscle testing score (MMT) than controls at the peak of muscle weakness [108.0 ± 55.0 vs 147.5 ± 22.0, p=0.008*]. In addition, patients with cardiac involvement more frequently had elevated creatine kinase (CK) throughout follow-up than controls [12/12 (100.0%) vs 131/177 (74.0%), p=0.041*]. On the other hand, there were no significant differences concerning the highest serum CK level throughout follow-up [1522.5 ± 6053.0 vs 1300.0 ± 2555.0, p=0.866]. In addition, there were no statistically significant differences between groups for serum aldolase, myoglobin, or lactate dehydrogenase levels.

Patients with cardiac involvement more frequently had oesophageal [6/12 (50.0%) vs 33/207 (15.9%), p=0.009*] and lung [10/13 (76.9%) vs 68/216 (31.5%), p=0.001**] involvements compared to the control group. Skin involvement was globally similar between groups. However, subcutaneous oedema was more frequent in patients with cardiac involvement [6/13 (46.2%) vs 41/210 (19.5%), p=0.034*]. Additionally, weight loss was more frequent in patients with cardiac involvement [6/12 (50.0%) vs 33/207 (15.9%), p=0.009*]. Joint, gastrointestinal and vascular involvements and the prevalence of cancer-associated myositis, dysphonia, and fever were not statistically different between groups.

Anti-SRP [3/11 (27.3%) vs 9/174 (5.2%), p=0.026*] antibodies were more commonly identified in patients with cardiac involvement. There were no statistically significant differences between groups regarding antisynthetase antibodies (individually or as a group) or any other MSA or MAA.

In the multivariate analysis, anti-SRP antibodies positivity (OR 104.3, 95%CI: 2.5-4277.8, p=0.014) was identified as a predictor of cardiac involvement in IIM patients, regardless of sex, ethnicity, age at diagnosis, and lung involvement. The remaining variables were not statistically significant predictors of cardiac involvement. However, only 81 patients were included in this model due to the high level of missing data on the ethnicity variable. In order to overcome this issue, a sensitivity analysis was performed, excluding this variable from the model. A model without ethnicity included 159 patients and also endorsed anti-SRP antibodies as independent predictors of cardiac involvement in IIM patients (OR 6.0, 95%CI: 1.1-31.8, p=0.035). In the latter model, lung involvement was also a predictor of cardiac involvement (OR 5.0, 95%CI: 1.2-21.7, p=0.031). Additionally, we also confirmed anti-SRP association with cardiac involvement (OR 19.4, 95%CI: 1.8-206.0, p=0.014) in a multivariate model with sex, age at diagnosis, lung involvement and minimum MMT8 as covariates. In this latter model, the remaining variables were not statistically significant predictors of cardiac involvement. A model including both ethnicity and minimum MMT8 was not used because the high number of patients missing data on these two variables leads to a statistical model with an overwhelmingly small number of included patients.

## Discussion

According to the literature, cardiovascular involvement in IIM patients ranges from 6-75%, depending on selection criteria, the definition of cardiovascular involvement, and the methods used to detect it (4, 16). The prevalence of cardiac involvement in our cohort was on the lower end of this spectrum. However, we should emphasise that we only considered cardiac involvement, excluding other types of vascular involvement (**Table 2**). This was a critical methodological decision that was pre-defined in the project protocol because vascular involvement is very loosely defined and could include manifestations such as Raynaud’s phenomenon, which is very prevalent in IIM and was not the focus of our work. Additionally, our study used real-world data from a national registry, so we cannot ensure that all patients were systematically screened for extramuscular involvement, which might have allowed for the underdiagnosing of subclinical cardiac involvement. Of note, all patients were followed in Rheumatology departments in tertiary centres. The aforementioned factors may have contributed to the arguably low prevalence of cardiac involvement in the Reuma.pt/Myositis cohort. Another factor that may have contributed is the recently improved IIM diagnostic capacity, for which we have a set of autoantibodies that help diagnose patients early in their disease course. Considering these biomarkers are recent, they were probably not available at the time of the previous studies regarding the prevalence of cardiac involvement in IIM. Therefore, our cohort may include more patients diagnosed and treated early who did not develop clinically apparent heart disease.

**Table 2:** Characteristics of the patients with cardiac involvement.

IIM subtype	Immunologic profile	Cardiac involvement
DM	anti-MDA5	56-year-old male presented with acute pulmonary oedema, with troponin levels 3-fold over the superior limit of the reference range (SLRR) and increased NT-proBNP at a maximum level of 2688 pg/mL; despite optimal diuretic therapy, the patient needed supplementary oxygen; echocardiogram showed center ventricle hypertrophy and diffuse hypokinesia, associated with severely compromised global systolic center ventricle function; CT scan also showed cardiomegaly, pericardial effusion and lung oedema; the patient was started on cyclophosphamide and prednisolone (1 mg/Kg/day), with significant clinical improvement and normalization of NTproBNP; cardiac MRI was performed after treatment onset and showed center ventricle hypertrophy with moderate to severe systolic function compromise; no coronary lesions were found on the coronary angiography
IMNM	anti-SRP	50-year-old female complaining of fatigue had an echocardiogram showing hypokinesia of the basal and medium segments of the inferior septal wall and a CT scan with mild pericardial effusion; EKG was normal; blood workup showed a slight increase of NTproBNP and troponin over 500-fold SLRR; cardiac MRI showed late enhancement of the subendocardium with hypokinesia of the same segment, as well as generalised myocardial oedema
DM	ANA, NXP2	30-year-old male presented with fatigue and had a normal echocardiogram and EKG; a blood workup showed a normal NTproBNP and raised levels of troponin more than 30-fold SLRR; cardiac MRI was performed and showed fibrotic changes with evidence of probable myocarditis sequela and a mild pericardial effusion
DM	ANA, Jo1	55-year-old female presented with dyspnea on exertion and pleuritic thoracic pain; blood workup showed a normal NTproBNP and troponin 100-fold SLRR; cardiac MRI showed showing late enhancement and pericardial effusion, confirming the suspected myocarditis and pericarditis
PM	ANA, anti-SRP	70-year-old female presented with dyspnea on exertion and raised troponin 10-fold SLRR; there were no relevant abnormalities on EKG or echocardiogram; CT scan showed a hypodensity of the inferior septal wall of the center ventricle’s apex with mild bilateral pleural effusion; coronary angiography was normal; a diagnosis of myocarditis was assumed, and the patient was treated with oral prednisolone (1 mg/Kg/day), intravenous immunoglobulin and cyclophosphamide, with significant clinical improvement; cardiac MRI was performed one month after the first cyclophosphamide pulse and showed a dilated center atrium but no enhancement
PM	ANA, anti-OJ	63-year-old male presented with fatigue and thoracalgia; the patient was diagnosed with pericarditis, based on the compatible clinical findings, tachycardia on EKG and considerable pericardial effusion on echocardiogram
PM	ANA, Jo1	66-year-old female complained of dyspnea with minimal efforts; troponin was 5-fold SLRR; EKG showed a right bundle branch block; the echocardiogram showed a dilated center side heart chambers; coronary angiography showed a major occlusion of the first diagonal (90%) and the anterior descending (>50%) coronary arteries
OS	ANA, Jo1	53-year-old female presented with palpitations and oppressive chest pain; tachyarrhythmia was noted; EKG and Holter showed supra-ventricular paroxysmic tachycardia with very frequent supraventricular extrasystoles; echocardiogram had a mild pericardial effusion
NM	ANA, EJ	64-year-old male complained of dyspnea and fatigue for increasingly smaller efforts; on EKG, there was a first-degree atrioventricular block, a complete right bundle branch block and a center anterior fascicular block; CT scan showed cardiomegaly and findings that suggested right cardiac failure; echocardiogram found a severely dilated center ventricle with compromised systolic function as well as a compromised systolic function of the right ventricle; the patient died despite the assisting team’s efforts
DM	ANA, anti-Mi-2	59-year-old female presented with palpitations; EKG and Holter showed frequent supraventricular extrasystoles as well as a complete center bundle branch block; no relevant abnormalities were found on the echocardiogram; CT scan did not show cardiomegaly
CADM	SSA	72-year-old female with severe bradycardia on EKG and a mildly dilated center atrium on echocardiogram
PM	ANA, Jo1	52-year-old male with no major cardiovascular risk factors presented with thoracalgia and palpitations; EKG showed new-onset atrial fibrillation; coronary angiography showed a massive occlusion (>70%) of the second circumflex coronary artery
IMNM	ANA, anti-SRP	42-year-old female with complaints of peripheral oedema, thoracalgia and dyspnea on exertion; troponin was 460-fold SLRR; EKG had nonspecific cardiac repolarization abnormalities; CT scan and echocardiogram revealed pericardial effusion as well as concentric hypertrophy of the center ventricle; cardiac MRI confirmed increased center ventricle wall thickness and could not exclude myocarditis; after treatment with intravenous methylprednisolone followed by oral prednisolone (1 mg/Kg/day), intravenous immunoglobulin and cyclophosphamide, the patient showed clinical improvement, troponin decreased significantly, and echocardiogram normalized

M, male; F, female; DM, dermatomyositis; IMNM - immune-mediated necrotizing myopathy; PM, polymyositis; NM, nonspecific myositis; OS, overlap syndrome; CADM, clinically amyopathic dermatomyositis; ANA, anti-nuclear antibodies; MDA5, melanoma differentiation-associated gene 5; SRP, signal recognition particle; NXP2, nuclear matrix protein 2; Jo1, histidyl tRNA synthetase; OJ, isoleucyl tRNA synthetase; EJ, glycyl tRNA synthetase; SSA, anti, Sjögren’s syndrome-related antigen A.

MSA and MAA have a prognostic influence on IIM, and different autoantibodies have been associated with different clinical characteristics (17–19). Although these associations have been well-established for lung, skin, or muscular manifestations, the same does not apply to cardiovascular manifestations.

There is some evidence in the previously published literature that there may be an association between anti-SRP and cardiac involvement in IIM, particularly cardiac disease-related symptoms and electrocardiographic and echocardiogram changes (12, 13). However, this association has been inconsistently reported (10, 11), with some studies suggesting a link between the positivity for this antibody and ILD but not cardiac manifestations (20). Recent evidence suggests that anti-SRP antibodies may have a pathogenic role targeting skeletal muscle (21, 22), with muscle fibre necrosis being confirmed in mice with transferred anti-SRP (23). Whether this contributes to cardiac involvement remains unclear. This background information helped recognise the likely association between anti-SRP antibodies and cardiac involvement in the univariate analysis as meaningful. Furthermore, the possibility of ILD being a confounder for this association (20) highlighted the need for including lung involvement in any multivariate analyses built to test anti-SRP association with cardiac involvement. In our cohort, anti-SRP antibodies were independently associated with cardiac involvement, regardless of sex, ethnicity, age at diagnosis, and lung involvement. This association was challenged by different multivariate models but was highly consistent in all of them.

This work supports previous reports of the association between the positivity of anti-SRP antibodies and cardiac involvement in IIM patients. Moreover, we attested to this association independently of lung involvement and further confirmed it through sensitivity analysis. These results were obtained in a South European cohort with a large representation of Caucasian patients, and additional validation in multiethnic cohorts is needed. Further research using large international cohorts, such as MyoNet, may potentially identify other clinical biomarkers that can help stratify the risk of cardiac involvement in IIM patients. In the meantime, considering our findings, we suggest a closer screening for heart disease in anti-SRP-positive patients.

## Data availability statement

The original contributions presented in the study are included in the article/supplementary material. Further inquiries can be directed to the corresponding author.

## Ethics statement

The studies involving human participants were reviewed and approved by Ethics Committee of Centro Académico de Medicina de Lisboa (195/21). The patients/participants provided their written informed consent to participate in this study.

## Author contributions

All authors contributed to the follow-up and recruitment of patients as well as data insertion. All authors re-evaluated their own cases of cardiac involvement. ED, MB and RC-M developed the project. MB and ED did the statistical analysis and wrote the manuscript. All authors contributed to the article and approved the submitted version.
